# An Account of the Remittent Fevers of *Cleveland, Ohio*

**Published:** 1839-09-21

**Authors:** George Mendenhall

**Affiliations:** Cleveland, Ohio


### AN ACCOUNT OF THE REMITTENT FEVERS *of Cleveland, Ohio.* By George Mendenhall, M. D.

To the Editors of the Medical Examiner.

Gentlemen,—In No. 34 of the Examiner, I notice a short account of the diseases prevailing in your city, and an invitation to distant correspondents to communicate the result of their observations. In accordance with this invitation, I send you the following, hastily drawn up, to use as you may think proper.

Cleveland, as you know, is situated on the southern shore of Lake Erie, at the northern termination of the Ohio canal. The town itself is principally on high ground, ninety feet above the level of the lake and Cuyahoga river. The river, which bounds the city on its western and southwestern limits, is very sluggish for about ten miles from its mouth. It has its rise from small lakes at and near the summit, dividing the waters of Lake Erie from the Ohio river. Those lakes are the receptacles of a large quantity of partly decomposed vegetable matter. The valley, near the mouth, is rather narrow and marshy; the latter is owing principally to the rise and fall of the river, created by the variation in the lake winds, and corresponds in some measure in effect with your tides on the Delaware and Schuylkill. From this you may premise that we are somewhat subject to remittents and intermittents in the summer and autumn, with the usual attending derangements of the viscera. The place is, upon the whole, healthy; the amount of sickness is mostly crowded into the months of August, September, and October, while the other months are very healthy. During the excavation of the Ohio canal in 1827 and 1828, the autumnal fevers were very severe and fatal. Of their particular character, I cannot speak from personal observations. For the four years preceding the present one, the autumnal remittents and intermittents have been characterized by a slightly inflammatory diathesis, evident hepatic congestion in all cases; and when protracted, a local determination to some one vital organ, most generally the brain, sometimes to the bowels, more seldom, but occasionally to the lungs. The indications of treatment were generally very plain. In the first stages the lancet was mostly called for once, seldom oftener. Active cathartics following the use of mercurials, were strongly indicated, and found of immense advantage. A very common prescription was

℞. Pulv. Dov. gr. viii.
Hyd. Chlor. Mit. gr. ii.

One every three hours during the night, and followed by a cathartic the following morning. This was generally successful in producing a healthy state of secretion from the bowels, determining to the surface, and producing a quiet, easy night's rest, when too much determination to the head did not exist. In that case, the nitrate of potash and tartar emetic were substituted for the Dover powders.

This treatment, continued for a few days, produced a more perfect remission or intermission, which called for the employment of quinine. Frequently the quinine could be used alone, but in other cases it became necessary to combine it with some diaphoretic, such as Dover's powder, or spts. nit. dulc. I think I have cut short many cases of remittent by combining the quinine with diaphoretics, which would have run a much longer course without its use, and when its administration alone would have been productive of evil consequences. This season, thus far, has offered no prevailing disease other than is usual to this climate and locality. The *particular character* of the disease, however, in the present season, is somewhat different.

Inflammatory symptoms prevail in a móre marked manner, and almost every case appears to be benefited by the use of the lancet once, and sometimes two or three bleedings are required. The course of the fever is more irregular. The intermissions and remissions occur at all hours, without regularity. Sometimes slight chills several times a day, while the skin is hot, and not followed by that kind of perspiration which attends the regular paroxysms. The liver congested and torpid, and occasionally, even in the first stages, tenderness of the bowels, particularly over the region of the ileo-cœcal valve. The pulse varies exceedingly at different hours of the day. In some cases, at one time of the day, venesection and tart. ant. are indicated; while at another time of the same day, you have to give diffusible stimulants. The treatment best suited is what I would call *an equalizing one*, which has to be adapted to the condition of the patient at different times,—deplete during the paroxysms of fever, and support during the remissions and intermissions. The brain is the organ most liable to suffer during the paroxysm of fever. If an active course of bleeding, cathartics, and diaphoretics is pursued at the commencement, the disease generally yields so as to bear the use of tonics; but greater caution is necessary in its use than in the fevers of preceding years. Occasionally, cases will continue for several weeks after the inflammatory symptoms of the first stages have subsided, without active depleting treatment. In other cases, local determinations to almost every tissue and organ of the body will be successively developed, and retard convalescence a long time, if it does not terminate fatally. If this state succeeds, you can neither deplete nor give tonics; your hands are in a great measure confined, and all that can be done is to follow up the local determinations as required at the time for each one, and wait for the recuperative energies of the system to effect a cure. On the contrary, I do not now recollect a case where this active course was adopted at the onset, which terminated in this manner. Out of some forty patients, I have lost one. This was one I did not dare to bleed, as I thought, on account of age; I was afraid she would not bear it. As it turned out, I was sorry the lancet was not used.

In addition to the above course, blisters and cups to the nape of neck, and to the abdomen, were frequently beneficial. In convalescence, tonics, used cautiously, were admissible. The prescription of blue mass and quinine was often necessary to cure and prevent visceral engorgments, and renewal of the attack.

*Cleveland, Ohio*, Sept. 16, 1839.

